# Exploring the relationship of triglyceride to high-density lipoprotein cholesterol and the triglyceride-glucose index with white matter hyperintensities in individuals with type 2 diabetes: a cross-sectional analysis

**DOI:** 10.3389/fendo.2025.1639083

**Published:** 2025-11-25

**Authors:** Jun Pan, Juan Zheng, Hua Dong, Haili Zhang, Zhiwei Hu, Zhangchao Tang, Hanwen Xu

**Affiliations:** Department of Endocrinology, The First People’s Hospital of Jiashan, Jiashan Hospital Affiliated to Jiaxing University, Jiaxing, Zhejiang, China

**Keywords:** type 2 diabetes mellitus, triglyceride glucose index, white matter hyperintensities, insulin resistance, triglyceride to high-density lipoprotein cholesterol ratio

## Abstract

**Background:**

Previous studies have shown that the triglyceride-glucose index (TyG) is associated with white matter hyperintensities (WMH) in a healthy population, acting as a marker of insulin resistance (IR). Triglyceride to high-density lipoprotein cholesterol ratio (TG/HDL-c) has been also recognized as marker for IR. However, the connection between TyG or TG/HDL-c and WMH in type 2 diabetes mellitus (T2DM) patients is unclear. The aim of this study was to examine the link between TG/HDL-c or TyG and WMH in patients with T2DM.

**Methods:**

The study enrolled a total of 420 T2DM patients. The WMH in baseline brain MRI scans was measured using the modified Fazekas scale. The relationship between WMH risk and severity in relation to TG/HDL-c and TyG was assessed through logistic and ordered logistic regression analyses. The variance inflation factor was employed to examine variable collinearity. Potential non-linear relationships between TG/HDL-c or TyG and the risk of WMH were examined using restricted cubic splines.

**Results:**

The median age of the study participants was 57.0 (18.0) years, 280 (66.7%) were men, and 263 (62.7%) were WMH-positive. The Fazekas score was correlated with the TG/HDL-c (r=0.366, P = 0.035) and TyG(r = 0.088, P = 0.025). Binary logistic regression analysis showed that TG/HDL-c (OR = 1.252, 95% CI 1.074-1.459) and TyG (OR = 1.883, 95% CI 1.359-2.609) were associated with the WMH. Multivariate adjusted restricted cubic spline plots showed a linear relationship between TG/HDL-c or TyG (P < 0.05, P-nonlinear > 0.05) and WMH. Multiple ordered logistic regression analyses showed that TG/HDL-c(OR = 1.123, 95% CI 1.028-1.226) and TyG (OR = 1.606, 95% CI 1.237-2.085) were independently associated with the burden of WMH. Subgroup analysis showed that TG/HDL-c were more substantially correlated with the WMH in male T2DM patients with a shorter duration of diabetes, younger age, lower blood pressure levels, and poor glycemic control (p<0.05). A comparable association of the TyG with WMH was also observed in these subgroups.

**Conclusion:**

The TyG and TG/HDL-c were independently and significantly associated with a higher prevalence and burden of WMH in patients with T2DM, which emphasizes the potential usefulness of these markers in early risk stratification.

## 1 Introduction

White matter hyperintensity (WMH) is an essential marker on radiographs for detecting small vessel disease in the brain (1). A previous study has shown that moderate or severe WMH at baseline is associated with an increased risk of dementia (117%), impairment in cognitive abilities (129%), functional impairment (121%), any recurrent stroke (65%), recurrent ischemic stroke (90%), all-cause mortality (72%), and cardiovascular mortality (102%) (2). Earlier research has indicated that WMH is associated with abnormalities in the cerebral microvasculature, and insulin resistance (IR) may be a crucial underlying mechanism for WMH.

Lipid and glucose metabolism disorders are commonly observed in patients with IR which is characteristic of many metabolic diseases, including hyperglycemia, hypertriglyceridemia and low high-density lipoprotein (3, 4). However, quantifying IR presents a significant challenge in clinical and research settings, primarily because of the complicated nature of its underlying processes and the absence of a universally accepted standard for measurement. Although the hyperglycemic clamp technique is acknowledged as the gold standard for evaluating insulin resistance, it is inappropriate for routine clinical use due to its invasiveness, technical complexity and the resources it requires (5). As emerging biomarkers, the triglyceride-glucose index (TyG) and triglyceride to high-density lipoprotein cholesterol ratio (TG/HDL-c) are used as alternative measures for IR, with their clinical relevance gaining traction in the Chinese population, which comprises aspects related to fat and blood glucose. The relationship between TyG and markers of adiposity, metabolism, and atherosclerosis associated with IR demonstrated moderate agreement with the hyperglycemic clamp (6). Furthermore, TyG showed marginally improved performance compared to the HOMA2-IR index. The TG/HDL-c is frequently utilized as a substitute marker for assessing IR. In the general population, there appears to be a direct link between the TG/HDL-c ratio and IR (7, 8).

To the best of our knowledge, IR and dysfunction of islet β-cells are the primary pathophysiological contributors to T2DM (9). As reliable indicators of IR, numerous studies have shown a significant link between TyG or TG/HDL-c and pre-diabetes/diabetes among Chinese individuals (10–12). As type 2 diabetes mellitus (T2DM) becomes more prevalent, the proportion of WMH linked to diabetes increases annually (13). WMH in patients with T2DM tend to be more irregular in shape and to increase in volume, and T2DM exacerbates changes in WMH shape and volume (14, 15). Furthermore, brain diseases such as stroke, dementia, and depression are increasingly being recognized as significant clinical complications of T2DM. Previous research data show that T2DM is associated with a 2.5-fold higher risk of ischemic stroke, a 1.5-fold higher risk of hemorrhagic stroke, and a 1.5-fold higher risk of dementia (13). These illnesses were also linked to WMH, and the presence of diabetes alongside WMH might worsen these conditions.

Since T2DM heightens the risk of WMH, it is necessary to identify the high-risk group early (16). Retrospective studies have shown that the TyG is correlated with a higher prevalence and greater load of WMH in a populations without diabetes (17, 18). A study using population-based MRI indicates that low levels of HDL-c might be linked to WMH in elderly individuals residing in rural China (19). And TG/HDL-c is connected to a higher occurrence and greater impact of cerebral WMHs in CSVD, which highlights a significant relationship between TG/HDL-c and WMHs (20). TyG and TG/HDL-c are markers of IR, which may represent an underlying mechanism of WMH. The TyG and the TG/HDL-c are more accessible and cost-effective compared to HOMA-IR and euglycemic-hyperinsulinemic clamp tests. The connection between TyG or TG/HDL-c and WMH in T2DM patients, who are more susceptible to WMH, is unclear. The purpose of this research was to investigate how TyG and TG/HDL-c relate to the risk and severity of WMH in T2DM patients.

## 2 Methods and materials

### 2.1 Study population

**Figure 1:**
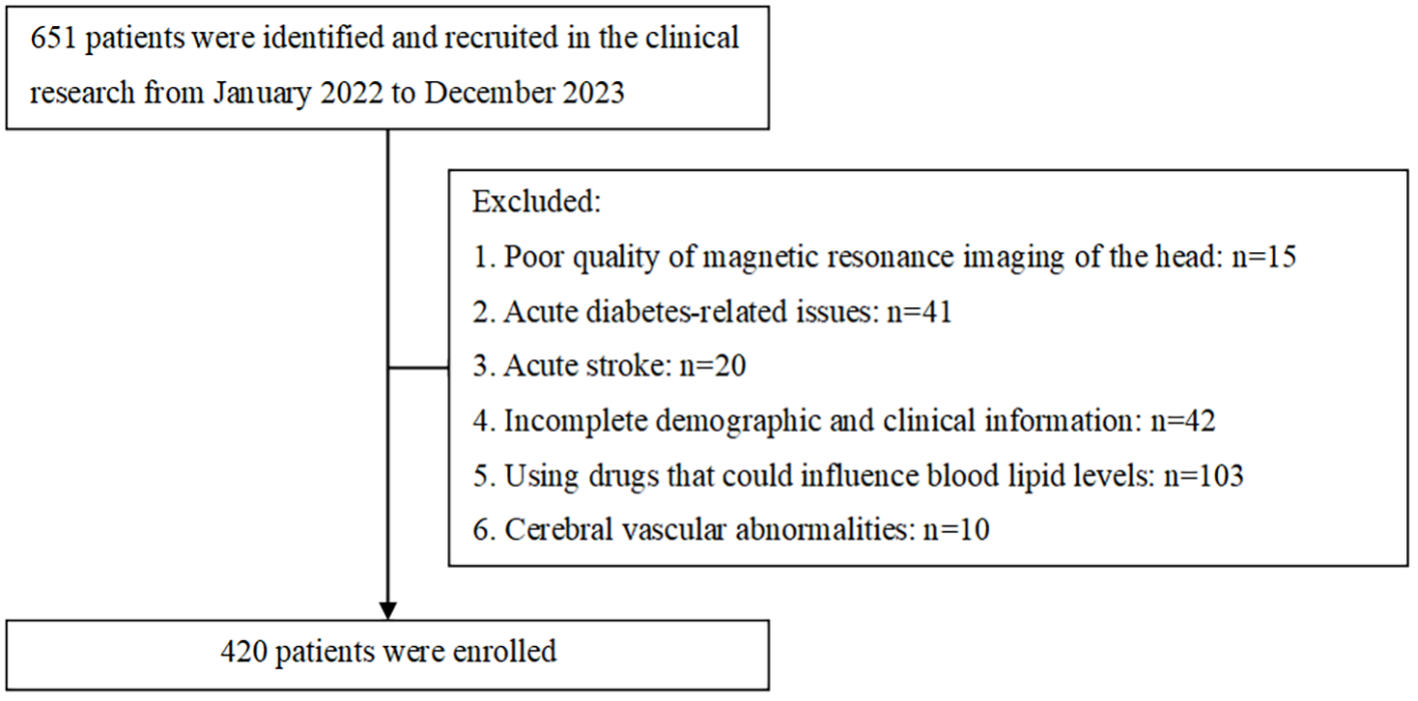
Study recruitment profile.

The sample size was determined assuming an expected exposure rate of 50% in the control group and an odds ratio of 2.0, with α = 0.05, power = 0.8, one controls per case, the sample should consist of 137 cases and 137 controls. Sample size estimation was conducted using R website for epi package (https://zstats.medsta.cn/samplesize/). In total, this study involved 420 T2DM patients confirmed (**Figure 1**), including 263 positive patients and negative 157 patients, which exceeded 137 per group. All patients were performed by Brain MRI scans on a 1.5T MR scanner (GE Signa VH/I, USA). Data that was missing, amounting to less than 10%, has been deleted outright. The imaging sequences included axial T1-weighted, T2-weighted, Fluid-Attenuated Inversion Recovery, and diffusion-weighted imaging.

Requirements for inclusion: 1. Age must be 18 years or older; 2. T2DM diagnosis according to the 2020 ADA Standards of Medical Care; 3. Possession of complete clinical records.

Criteria for exclusion: 1. Acute diabetes-related issues, including hyperosmolar hyperglycemia, acute infections, and ketoacidosis; 2. Consumption of lipid-modifying drugs like statins, fibrates, or other medications that might significantly alter lipid results; 3. Severe dysfunction with the liver, kidneys, or other organs, severe infections, malignant tumors, or other critical illnesses; 4. Lack of general and pertinent clinical data; 5. Women who are pregnant or breastfeeding; 6. Cerebrovascular abnormalities, intracranial lesions, or other conditions affecting cerebral blood vessels that may alter the interpretation of WMH.

### 2.2 Clinical evaluation

Participants’ demographic characteristics and medical histories were reviewed, focusing on hypertension, age, gender, and the duration of their diabetes. Blood glucose, blood pressure, and serum lipid levels were evaluated on the second day of admission from venous blood samples collected after fasting overnight. HbA1c was assessed by employing high-performance liquid chromatography with the hemoglobin testing system from Bio-Rad Laboratories, based in Hercules, CA, USA. Plasma glucose was measured using glucose oxidase, while total cholesterol (TC), high-density lipoprotein cholesterol (HDL-c), triglyceride (TG), and low-density lipoprotein cholesterol (LDL-c) were assessed with the autoanalyzer (AU5800; Beckman coulter, CA, USA). All assessments were performed in the same laboratory, and the TG/HDL-c was calculated as TG (mmol/L) divided by HDL-c (mmol/L) (21). The TyG = ln[1/2*TG(mg/dl) × fasting blood glucose (mg/dl)] (22). TG:1 mmol/L(88.5 mg/dL), fasting blood glucose:1 mmol/L(18.0 mg/dL).

### 2.3 WMH rating scale

WMH extent was assessed using FLAIR images, and the disease was categorized into grades 0–3 with a modified Fazekas score (23). The Fazekas score was independently evaluated by a physician who had received training and certification from a senior neuroradiology expert and was unaware of any clinical or laboratory data. Another senior neurologist, who was independent and highly experienced, assessed the scoring results. The agreement between the two Fazekas scores was determined by the weighted kappa coefficient (0.99). The Fazekas grading system classifies WMH as follows: Fazekas grade 0 indicates the absence of WMH. Fazekas grade 1 is characterized by WMH presenting as caps and/or a thin lining around the lateral ventricles, and/or punctate foci within the deep grey matter. Fazekas grade 2 is defined by WMH forming a smooth halo around the ventricles and exhibiting either larger or beginning confluent foci in the deep white matter. Finally, Fazekas grade 3 is identified by irregular periventricular signals extending into the deep white matter and/or confluent WMH foci within the deep white matter. The participants were categorized into those with WMH (Fazekas grade ≥1) and no-WMH group.

### 2.4 Statistical analysis

SPSS 25.0 (IBM Corp, Armonk, NY, USA) was employed for statistical analysis. Continuous variables were expressed as medians and interquartile ranges (IQR), and categorical variables were expressed as proportions. The Mann–Whitney U test was employed in our study to compare continuous variables, and Pearson’s χ2 test was used for categorical variables. In order to analyze the data, we conducted both logistic regression and ordered logistic regression analyses utilizing four distinct patterns. Pattern 1 was unadjusted; Pattern 2 was adjusted for age and sex; Pattern 3 was further adjusted for the variables in Pattern 2, in addition to hypertension, duration of diabetes, and fasting blood glucose levels; and Pattern 4 included adjustments for the variables in Pattern 3, with the addition of glycated hemoglobin, systolic blood pressure, and diastolic blood pressure. To evaluate the collinearity assumption, variance inflation factors (VIFs) were calculated, with a VIF below 5 suggesting no significant collinearity. The calibration of our model was evaluated by Hosmer–Lemeshow test (HL test) and test of parallel lines. The association between TyG or TG/HDL-c and WMH risk was illustrated using a restricted cubic spline regression line, performed with R software (version 3.6.3, RMS package, knot=4)). The odds ratio (OR) and the 95% confidence interval (CI) were computed, considering a P value <0.05 as statistically significant.

## 3 Results

### 3.1 Features of the study group

Between January 2022 and December 2023, 651 participants were screened. Participants with poor-quality of magnetic resonance imaging of the head (n = 15), acute diabetes-related issues (n = 41) or acute stroke (n = 20), incomplete demographic and clinical information (n=42), using drugs that could influence blood lipid levels (n=103) and cerebral vascular abnormalities (n=10) were excluded. The study included 420 individuals. The median age for the population was 57.0(18.0) years, and 280 (66.7%) of the individuals were male. There were 157 patients with no WMH (Fazekas score = 0), 263 patients with positive WMH (Fazekas score≥1) among whom 209 (49.8%) participants were Fazekas grade = 1, 39 (9.3%) participants were Fazekas grade = 2, 15 (3.6%) participants were Fazekas grade = 3. (**Table 1**).

**Table 1:** The demographic and clinical characteristics of all participants.

	Characteristics (N = 420)
Male, n (%)	280 (66.7)
Age (year)	57.0 (18.0)
Height (cm)	167.0 (12.0)
Weight (kg)	71.0 (16.0)
BMI (kg/m2)	24.9 (4.0)
SBP (mmHg)	135.0 (25.0)
DBP (mmHg)	81.0 (14.0)
Duration of diabetes (years)	5.1 (8.0)
FINS (μIU/ml)	4.1 (5.9)
FCP (nmol/L)	1.3 (1.0)
TG (mmol/L)	1.5 (1.0)
HDL-c (mmol/L)	1.1 (0.3)
TC (mmol/L)	4.4 (2.0)
LDL-c (mmol/L)	2.6 (1.0)
NHDL (mmol/L)	3.4 (2.0)
FBG (mmol/L)	6.4 (1.5)
Hypertension, n (%)	204 (48.6)
TyG index	8.2 (1.0)
TG/HDL-c	1.5 (1.0)
HbA1c (%)	9.4 (3.0)
Fazekas grade of WMH	
0 (n, %)	157 (37.3)
1 (n, %)	209 (49.8)
2 (n, %)	39 (9.3)
3 (n, %)	15 (3.6)

Information is provided as median (IQR) or as a number (percentage).

BMI (body mass index), SBP (systolic blood pressure), DBP (diastolic blood pressure), FINS (fasting insulin), FCP (fasting c-peptide), TG (triglycerides), HDL-c (high-density lipoprotein cholesterol), TC (total cholesterol), LDL-c (low-density lipoprotein cholesterol),NHDL (nonhigh-density lipoprotein cholesterol), FBG (fast blood glucose).

### 3.2 Correlations between clinical characteristics and the Fazekas score

Spearman’s correlation analysis was employed to analyze the connection between the Fazekas score and the TyG or TG/HDL-c. According to **Table 2**, there was a considerable positive correlation between the severity of WMH and factors such as Age, Duration of diabetes, TyG, FBG, SBP, and TG/HDL-c (P < 0.05). The severity of WMH was negatively correlated with DBP (P < 0.05). Therefore, when we adjusted for the effects of crucial influences that could confound outcomes, the TyG and TG/HDL-c were independently and significantly associated with burden of WMH. Logistic regression analysis also suggested the same results (**Table 3**).

**Table 2:** Correlation between clinical markers and the Fazekas score.

Variables	R value	P value
Age	0.544	<0.001
TyG	0.088	0.025
FBG	0.137	<0.001
SBP	0.200	<0.001
DBP	-0.082	0.037
TG/HDL-c	0.366	0.035
HbA1c	0.003	0.941
Duration of diabetes	0.225	<0.001

The p-value was determined through Spearman correlation analysis, where the r value indicates Spearman's correlation coefficient. P-value < 0.05 suggests a statistically significant difference.

**Table 3:** The odds ratios and 95% confidence intervals for TG/HDL-c and TyG in relation to WMH severity.

	TG/HDL-c		TyG	
	OR (95% CI)	P	OR (95% CI)	P
Pattern 1	1.121 (1.029, 1.220)	0.009	1.400 (1.135, 1.728)	0.002
Pattern 2	1.188 (1.091, 1.294)	<0.001	1.958 (1.537, 2.494)	<0.001
Pattern 3	1.130 (1.036, 1.234)	0.006	1.654 (1.278, 2.140)	<0.001
Pattern 4	1.123 (1.028, 1.226)	0.010	1.606 (1.237, 2.085)	<0.001

The odds ratios (OR) and 95% confidence intervals (CI) were derived using ordered logistic regression. Model adjustments consistent with **Table 4**.

### 3.3 Associations of the TG/HDL-c and TyG with the risk of WMH

**Figure 2:**
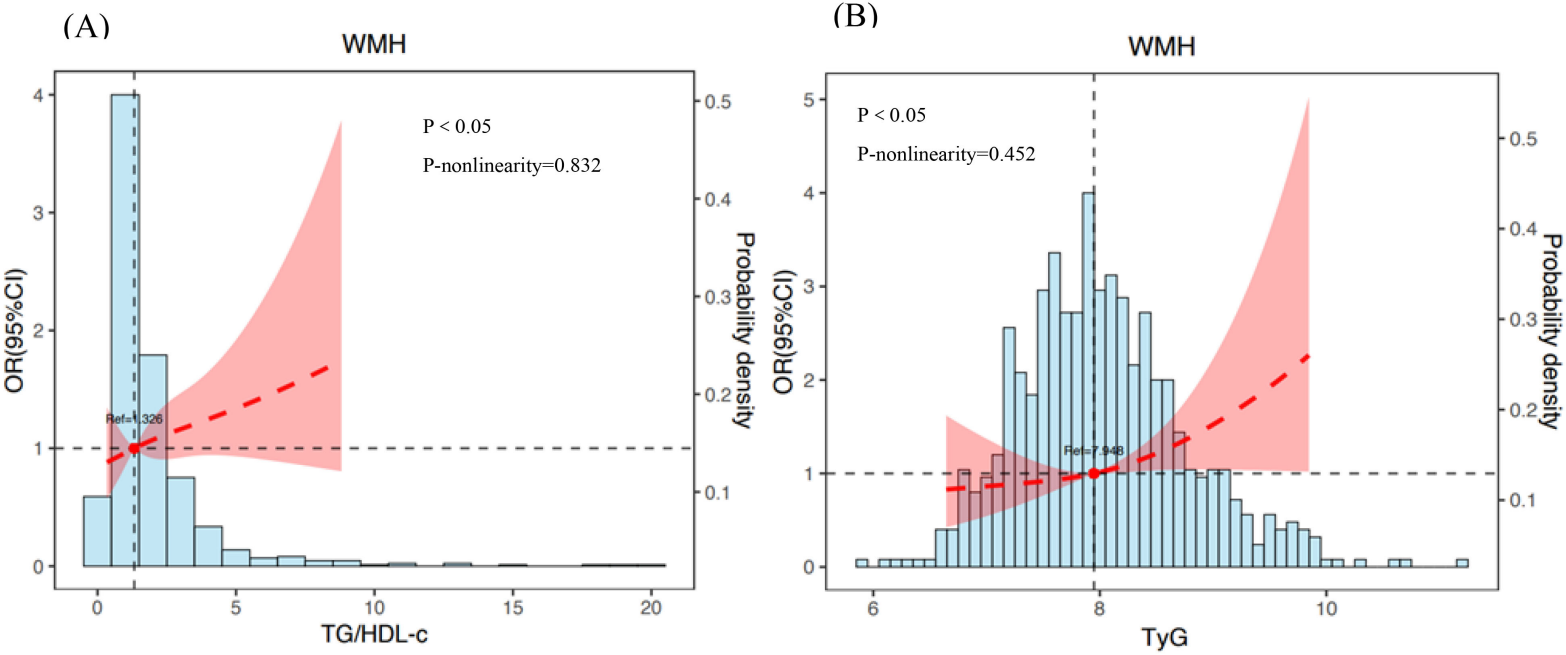
The association between TG/HDL-c **(A)** or TyG **(B)** and risk of WMH. Adjusted for variables such as age, sex, diabetes duration, hypertension, duration of diabetes duration, FBG, glycated hemoglobin, SBP and DBP. The dotted line indicates odds ratio, while the shadow indicates 95% CI.

**Table 4** illustrates the connection between the TG/HDL-c or TyG and the occurrence of WMH using logistic regression patterns. Following further adjustment for confounding variables in pattern 4, there was still a correlation between TG/HDL-c and WMH (OR 1.252, 95% CI 1.074-1.459, P = 0.004). In the fully adjusted pattern 4, a meaningful link between the TyG index and the prevalence of WMH was also noted (OR 1.883, 95% CI 1.359-2.609, P < 0.001). Similarly, after controlling important factors, the application of restricted cubic spline regression revealed that the TG/HDL-c was correlated with an increased risk of WMH. The relationship exhibited a significant deviation from linearity(P <  0.05, p-nonlinearity = 0.832, **Figure 2**). Additionally, a noteworthy linear association was identified between the TyG and the risk of WMH (P <  0.05, p-nonlinearity = 0.452, **Figure 2**). The analysis for collinearity diagnostics revealed that the VIFs of the risk factors were less than 5, indicating no strong multicollinearity among the variables. The HL test showed that our predicted and observed values are close (P > 0.05). Residual analysis indicated that the fundamental assumptions of the model are satisfied.

**Table 4:** Odds ratios and 95% confidence intervals for the TG/HDL-c and TyG associated with the risk of WMH.

	TG/HDL-c		TyG	
	OR (95% CI)	P value	OR (95% CI)	P value
Pattern 1	1.121(1.004,1.252)	0.043	1.544(1.208,1.972)	0.001
Pattern 2	1.356(1.164,1.579)	<0.001	2.235(1.655,3.018)	<0.001
Pattern 3	1.260(1.084,1.464)	0.003	1.941(1.409,2.673)	<0.001
Pattern 4	1.252(1.074,1.459)	0.004	1.883(1.359,2.609)	<0.001

The odds ratios (OR) and 95% confidence intervals (CI) were derived using logistic regression. Pattern 1 was unadjusted; Pattern 2 accounted for age and sex; Pattern 3 included adjustments for hypertension, duration of diabetes duration, and FBG along with Pattern 2; Pattern 4 included adjustments for glycated hemoglobin, SBP and DBP with Pattern 3.

### 3.4 Stratified analysis for associations of the TG/HDL-c and the TyG with the risk of WMH

The relationship was analyzed in a stratified way according to potential modifiers (**Table 5**). For female T2DM participants aged 60 or older, with HbA1c less than 7%, and SBP of 130 mmHg or more, and a diabetes history of more than 5 years, there were no significant associations between TG/HDL-c and WMH risk, which might be attributed to the very small sample size. A similar relationship between TyG and WMH risk was also noted in these subgroups. The stratified analysis did not reveal any interactions.

**Table 5:** Subgroup studies on the association of TG/HDL-c or TyG with the risk of WMH.

Subgroups	N	TG/HDL-c		TyG	
		OR (95% CI)	P	OR (95% CI)	P
Sex					
Male	280	1.238 (1.048, 1.462)	0.012	1.815 (1.256, 2.625)	0.002
Female	140	1.196 (0.819, 1.746)	0.353	1.927 (0.947, 3.921)	0.070
Age, years					
<60	239	1.407 (1.154, 1.715)	<0.001	2.390 (1.599, 3.574)	<0.001
≥60	181	1.023 (0.838, 1.248)	0.825	1.140 (0.632, 2.057)	0.663
HbA1c, %					
<7	52	1.701 (0.930, 3.109)	0.084	1.910 (1.341, 2.721)	<0.001
≥7	368	1.240 (1.054, 1.458)	0.009	1.902 (1.343, 2.694)	<0.001
Duration of diabetes, years					
<5	181	1.475 (1.138, 1.911)	0.003	2.578 (1.527, 4.352)	<0.001
≥5	239	1.094 (0.913, 1.311)	0.332	1.433 (0.918, 2.238)	0.113
SBP, mmHg					
<130	159	1.459 (1.078, 1.976)	0.014	2.281 (1.317, 3.952)	0.003
≥130	261	1.157 (0.966, 1.385)	0.112	1.604 (1.047, 2.455)	0.030

Considering age, sex, hypertension, the duration of diabetes, FBG, glycated hemoglobin, SBP and DBP. Using multivariable logistic regression models, the OR and 95% CI were assessed.

### 3.5 Relationships of the TG/HDL-c and TyG with the severity of WMH

**Table 3** presents the connection between the TG/HDL-c or TyG index and the severity of WMH using ordered regression models. Following additional adjustments for confounders, TG/HDL-c was still significantly associated with WMH (OR 1.123, 95% CI 1.028-1.226, P = 0.010). The TyG was significantly and positively related with WMH in a fully adjusted model (OR 1.606, 95% CI 1.237-2.085, P < 0.001). Stronger connections were found between the TyG and WMH compared to the TG/HDL-c. The VIFs indicating no strong multicollinearity among the variables. The test of parallel lines indicated that the proportional odds assumption was met (χ² = 24.109, df = 18, P = 0.151). Residual analysis indicates that the fundamental assumptions of the model are satisfied.

### 3.6 Stratified analysis for associations of the TG/HDL-c and the TyG with the severity of WMH

Additionally, stratified analysis of the relationship between the TG/HDL-c or the TyG index and the burden of WMH was performed according to the potential modifiers. In females aged 60 years, with HbA1c below 7%, and SBP of at least 130 mmHg, who have had diabetes for over 5 years, there was no significant link between TG/HDL-c and WMH severity. A similar relationship between TyG and WMH was also noted in these subgroups (**Table 6**). The stratified analysis did not reveal any interactions.

**Table 6:** Subgroup studies on how TG/HDL-c or TyG index relates to the severity of WMH.

Subgroups	N	TG/HDL-c		TyG index	
		OR (95% CI)	P	OR (95% CI)	P
Sex					
Male	280	1.140 (1.023, 1.269)	0.017	1.560 (1.139, 2.136)	0.006
Female	140	1.105 (0.923, 1.322)	0.276	1.756 (1.070, 2.880)	0.026
Age, years					
<60	239	1.229 (1.077, 1.402)	0.002	2.092 (1.446, 3.022)	<0.001
≥60	181	1.074 (0.943, 1.221)	0.284	1.252 (0.847, 1.850)	0.260
HbA1c,%					
<7	52	1.343 (0.896, 2.014)	0.154	1.919 (0.800, 4.604)	0.144
≥7	368	1.121 (1.023, 1.229)	0.014	1.632 (1.235, 2.158)	0.001
Duration, years					
<5	181	1.158 (1.029, 1.303)	0.015	1.891 (1.270, 2.818)	0.002
≥5	239	1.064 (0.925, 1.225)	0.385	1.354 (0.946, 1.935)	0.097
SBP,mmHg					
<130	159	1.170 (1.030, 1.328)	0.016	2.036 (1.328, 3.124)	0.001
≥130	261	1.088 (0.957, 1.236)	0.197	1.332 (0.951, 1.866)	0.095

Considering age, sex, hypertension, the duration of diabetes, FBG, glycated hemoglobin, SBP and DBP. Using multivariable ordered logistic regression models, the OR and 95% CI were assessed.

## 4 Discussion

T2DM is acknowledged as a major concern in public health, impacting both people’s lives and healthcare expenditures significantly. People suffering from T2DM face a notably increased chance of experiencing high signal intensity in WMH, which is a cerebral microvascular condition connected to dementia, cognitive issues, and stroke (2). Our research showed a significant positive correlation between TG/HDL-c or TyG and the heightened risk or severity of WMH in individuals with T2DM. The observed associations remained significant after adjusting for important confounding factors. However, the study employed a cross-sectional design, indicating that causal inferences should be approached with caution. Future longitudinal studies are planned to validate these findings and explore the underlying biological mechanisms.

The definitive cause of WMH is not well understood, but there is mounting evidence that insulin resistance is a critical underlying mechanism (24, 25). Patients with IR, apart from those with T2DM, are likely to develop hypertension and atherosclerosis (26). These are all risk factors associated with WMH. As a substitute marker for IR, the TyG index has been proposed recently and shows a good correlation with IR (27). We revealed that TyG is closely related to the risk and severity of WMH, which is consistent with the studies in a populations without diabetes (17, 18). Additionally, for those with T2DM, TG/HDL-c acts as a simple and reliable indicator of IR and is connected to a higher occurrence and impact of WMH. This finding is consistent with past research that highlights a significant relationship between TG/HDL-c and cerebral WMHs in CSVD (20). And after adjusting for confounding factors, our model has showed that TyG’s performance was slightly improved over TG/HDL-c in terms of OR values. TyG showed slightly better performance as indicators of IR compared to TG/HDL-c, aligning with findings from earlier research (28). However, further research is still needed.

There are several possible mechanisms that IR could account for the mechanism of WMH. One possible explanation could be related to the dysfunction of the blood-brain barrier (BBB). A characteristic of IR is the malfunctioning of the endothelium (29), featuring reduced nitric oxide bioavailability and increased endothelin-1 release, which may trigger endoplasmic reticulum stress, oxidative stress, mitochondrial dysfunction, and the activation of pro-inflammatory cytokines, resulting in the breakdown and functional suppression of the BBB (30, 31). The leakage of toxic substances into perivascular tissue due to BBB dysfunction can interfere with waste clearance by the glymphatic system, potentially causing the development and progression of WMH (32). Another mechanism that might be involved is the reduction of cerebral blood flow (CBF). IR can reduce nitric oxide levels and boost endothelin-1 release, leading to vasoconstriction and decreased blood flow to the brain (29). It has also been reported that reduced CBF might cause chronic hypoxia-ischemia in the brain, which is connected to the development of WMH and an increase in regional WMH volume (33).

Besides, arterial stiffness should be considered. Insulin resistance has been associated with arterial stiffness in a range of medical conditions according to earlier findings (34). As a newly described marker of cerebral vascular stiffness, global cerebral pulse wave velocity has been shown to correlate with WMH volume, suggesting that arteriosclerosis is associated with WMH (35). The stiffening of blood vessels leads to fluid accumulation in perivascular spaces and hampers interstitial flushing, playing a role in WMH. The associations between IR and cerebrovascular diseases, as well as the related risk factors, are relatively definite. Identifying high-risk WMH patients early can assist clinicians in quickly adopting rational strategies for treatment and prevention to slow down WMH progression.

The Rhineland Study has shown that the increase in WMH load accelerates with age (36). Our findings, similar to Rhineland Study, indicated that the age correlates with a higher risk and severity of WMH. WMH showed a strong association with both concurrent and historical elevated hypertension, with the greatest population burden of severe WMH attributed to SBP (37). Our investigation additionally demonstrates a strong affiliation between the SBP and WMH. Interestingly, this research found that WMH severity had a significant positive correlation with SBP, while showing a negative correlation with DBP. The contradictory nature of these result effectively illustrates the key role that large-artery atherosclerosis plays in the development of cerebral small vessel disease. The condition of arteriosclerosis results in higher SBP and pulse pressure, along with a decrease in DBP (38). Findings from studies on middle-aged and senior populations indicate that a greater difference in pulse pressure is connected to more severe WMH (39). This elevated pulse pressure influences cerebral microcirculation, causing damage to vascular endothelial cells and affecting the BBB (40). In individuals with atherosclerosis, the WMH in the brain simultaneously experience the effects of systolic hypertension and diastolic hypoperfusion ischemia, which together accelerate the progression of WMH degeneration.

Research from the past has suggested that among participants with diabetes, those with HbA1c ≥ 7.0% had an increased burden of WMH compared with those with HbA1c < 7.0% (41). The findings from our stratified analysis also support a stronger association between TyG or TG/HDL-c and WMH in patients with HbA1c ≥ 7.0%. Our findings indicate that TG/HDL-c or TyG has a stronger link to WMH in men compared to women. This could be due to variations in participant selection, and more studies are necessary to assess the gender differences in the association.

## 5 Strengths and limitations

The key finding of this research is that it is the first to demonstrate that novel IR could have a more substantial effect on the risk of WMH in T2DM. The results illuminate the possible role of lipid metabolism disorders in the emergence of WMH. The findings of this study facilitate early risk assessment for individuals with T2DM. However, our research and interpretations are subject to several limitations. Firstly, this study did not assess the precise volume of WMH utilizing the Software for Neuro-Image Processing in Experimental Research (42). Furthermore, the participants in this study were influenced by additional variables, including racial, habitual lifestyle factors (such as smoking, alcohol drinking and physical exercise) and clinical factors or medication use (such as antihypertensives, antiplatelets and antihypertensive classes), which might have a connection to WMH. And limitations in data collection hindered us from acquiring MRI data from non-diabetic patients, which was gathered from a single hospital and might restrict the applicability of the study’s findings. It is not clear if the sample accurately represents the wider diabetic population.

## 6 Conclusion

In summation, our study demonstrated that both the TG/HDL-c and the TyG are independently correlated with the risk and extent of WMH in patients with T2DM. This observation contributes essential knowledge for the early identification of risks and personalized medical care.

## Data Availability

The raw data supporting the conclusions of this article will be made available by the authors, without undue reservation.
